# Ex Vivo Quantitative Evaluation of Beam Hardening Artifacts at Various Implant Locations in Cone-Beam Computed Tomography Using Metal Artifact Reduction and Noise Reduction Techniques

**DOI:** 10.3390/diagnostics15243201

**Published:** 2025-12-15

**Authors:** Cengiz Evli, Merve Önder, Ruben Pauwels, Mehmet Hakan Kurt, İsmail Doruk Koçyiğit, Gökhan Yazıcı, Kaan Orhan

**Affiliations:** 1 Department of Oral and Maxillofacial Radiology, Faculty of Dentistry, Ankara University, 06560 Ankara, Turkey; 2 Department of Dentistry and Oral Health, Aarhus University, 98000 Aarhus, Denmark; 3 Department of Oral and Maxillofacial Surgery, Kırıkkale University, 71450 Kırıkkale, Turkey; 4 Department of Physical Therapy and Rehabilitation, Faculty of Health Sciences, Gazi University, 06680 Ankara, Turkey; 5 School and Hospital of Stomatology, Cheeloo College of Medicine, Shandong University, Jinan 250012, China

**Keywords:** artifact, cadaver, CBCT, MAR, AINO

## Abstract

**Purposes:** Beam hardening artifacts caused by dental implants remain one of the most significant limitations of cone-beam computed tomography (CBCT), often compromising the evaluation of peri-implant bone and potentially masking critical diagnostic findings. Although metal artifact reduction (MAR) and noise-optimization filters such as the Adaptive Image Noise Optimizer (AINO) are widely available in commercial CBCT systems, their effectiveness varies depending on implant configuration and scanning parameters. A clearer understanding of how implant positioning influences artifact severity—together with how MAR and AINO perform under different conditions—is essential for improving diagnostic reliability. **Materials and Methods:** A fresh frozen cadaver head, with dental implants inserted using two configurations (C1 and C2), was scanned using different scan parameters, with and without metal artifact reduction and image optimization filters. The percentages of gray value alteration due to artifacts were evaluated, using registered pre-implant scans as a control. Regions of interest were defined by an experienced researcher. For the two implant conditions, ROIs were placed as follows: C1—lingual, buccal and mesial to the mesial implant; lingual, buccal and distal to the distal implant; and an additional ROI between the implants (*n* = 7); C2—lingual, buccal, mesial and distal to each implant (*n* = 8). For each ROI, the mean gray value was measured in five consecutive axial slices, and rescaled according to calibration points in air and soft tissue. **Results:** Significant differences were found in gray values across configurations and scan modes. In the C2 configuration, combined MAR and AINO restored gray values in certain ROIs from 1.227 (OFF) to 1.223 (MAR+AINO), closely matching the control (1.227). In contrast, C1 showed limited improvement; for example, buccal ROI gray values decreased from 3.978 (OFF) to 3.323 (AINO) compared to the control (3.273), with no significant benefit from additional MAR. **Conclusion:** Artifacts from implants can be significantly affected by their (relative) position and the use of MAR and AINO.

## 1. Introduction

Cone-beam computed tomography (CBCT) devices have played an important role in diagnostics of the maxillofacial region [1]. CBCT devices have many advantages compared to multi-detector computed tomography (MDCT) such as a smaller physical footprint, lower radiation dose, lower cost, and a high, isotropic spatial resolution. However, as with MDCT, there are several sources of artifacts in CBCT, including scatter, noise, beam hardening, photon starvation, motion, and ring artifacts [2,3].

Beam hardening is one of the most prominent and common effects that cause artifacts. An X-ray spectrum consists of individual photons with various energies, with the maximum energy determined by the tube voltage (kV) and the mean (or effective) energy by the kV, filtration and anode properties. The beam becomes “harder” as it passes through an object, that is, its average energy increases, because primarily low-energy photons are absorbed as it passes through matter. Because conventional reconstruction algorithms assume a monochromatic beam, they cannot compensate for these variations in beam energy, which results in artifacts [4,5]. The nature of beam hardening artifacts depends on its severity, and can range from a center-to-edge gray value gradient (cupping) to dark/bright streaks [6,7,8]. When the beam has to pass through highly dense and/or multiple metal objects, only a minute fraction of the x-rays passes through. This effect is referred to as photon extinction, which typically manifests as dark regions adjacent to (especially in-between) the metal object. These artifacts are clinically relevant, as they occur in areas where there are structures such as metallic restorations, surgical splints, orthodontic brackets and dental implants, and may affect the image quality and prevent the clinician from noticing pathosis in the artifact area [5,6,9,10].

In the context of implant placement, evaluating the thickness and quality of the cortical and trabecular bone around the implant is critical in terms of primary stability and osseointegration, which can determine implant success. In addition, the early detection of bone defects, such as fenestrations and dehiscences around the implant also significantly affects its success. While CBCT can provides a 3D examination of bone quantity, bone quality and bone defects around implants, unlike intra-oral or panoramic radiography, the artifacts significantly affect the image quality, preventing the clinician from noticing potential issues at an early stage [11,12,13].

There are studies that explored the adaptation of scan parameters such as tube current, kilo-voltage, number of projections, field of view, and voxel size to reduce the artifacts that negatively affect the image quality [14,15,16,17,18,19]. Other studies have explored the use of Metal Artifact Reduction (MAR) algorithms and other methods such as Adaptive Image Noise Optimizer (AINO), which are currently found on several CBCT models. However, the effectiveness of these reconstruction modes at a diagnostic level is debatable [1,2,6,8]. There are studies in which the diagnostic accuracy of MAR is not very effective in reducing artifact in CBCT images [14,15,16,17,18,19]. AINO works by distinguishing noise from fine details in images; this is expected to increase overall image quality, which may lead to a diagnostic benefit [20]. Seeing as one of the effects of metallic objects is an increased (relative) noise level, especially in regions with low signal in the projection domain, it is possible that AINO affects the depiction of metal artefacts, especially when considering the combination with MAR.

The aim of this study is to evaluate how different implant configurations influence metal artifacts commonly observed in CBCT images, particularly those caused by dental materials. Specifically, we examine how implant placement affects the severity of beam hardening artifacts and assess the effectiveness of the Adaptive Image Noise Optimizer (AINO), both alone and in combination with the highest level of Metal Artifact Reduction (MAR), in minimizing these artifacts. We hypothesize that different implant configurations significantly affect artifact severity, and the application of AINO, either alone or combined with MAR, will more effectively reduce beam hardening artifacts compared to standard reconstruction modes.

## 2. Materials and Methods

### 2.1. Sample Preparation and Scanning

A fresh-frozen human cadaver head was utilized to closely replicate both hard and soft tissues as encountered in clinical imaging. The specimen belonged to a male individual in his fifth decade of life. The study protocol was reviewed and approved by the Ethics Committee of Gazi University (Approval Code: 2025-1710; Date: 9 October 2025). The cadaveric specimen had been preserved at −15 °C and was allowed to reach room temperature over a 24 h period prior to the imaging procedures.

**Figure 1:**
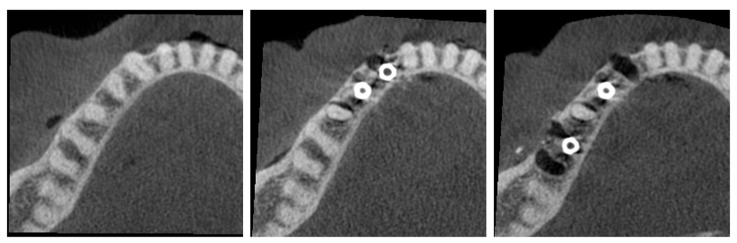
(**Left**) C0 configuration (no implants). (**Middle**) C1 configuration (adjacent implants). (**Right**) C2 configuration (distant implants).

Prior to implant placement, the cadaver underwent cone beam computed tomography (CBCT) scanning (C0; Figure 1). Following tooth extraction, pilot holes were drilled using the surgical kit of the Nucleoss brand implant system, with continuous saline irrigation for cooling. In single-rooted tooth regions, drilling was directed toward the root apex, whereas in multi-rooted areas, it was aimed at the center of the inter-radicular septum. Implant osteotomies were prepared to accommodate implants with a diameter of 4.1 mm and a length of 12 mm. In accordance with the manufacturer’s guidelines, drilling was performed at 35 Ncm torque and 20 rpm. Upon completion of the final drilling, the implants (Nucleoss T6, 4.1 mm diameter/12 mm length; NucleOSS, İzmir, Turkey) were placed into the osteotomy sites without irrigation. Implant placement was conducted in accordance with two predefined configurations (Figure 1):

- C1—Configuration where two implants are side by side, that is, there are no teeth between the implants.
- C2—Configuration with a tooth between the implants.

The C1 and C2 configurations were subjected to CBCT scanning using various imaging protocols. All scans were acquired with the ProMax 3D Max CBCT unit (Planmeca, Helsinki, Finland). The scan parameters were standardized across all acquisitions and included a field of view (FOV) of 130 × 55 mm, 96 kV tube voltage, 5.6 mA tube current, 12 s exposure time, and a voxel size of 0.2 mm. Reconstructions were performed for each configuration using the following proto-cols: (1) ‘OFF’—standard reconstruction without Metal Artifact Reduction (MAR) or Adaptive Image Noise Optimizer (AINO); (2) ‘MAR’—the highest available MAR level provided by the device was selected; (3) ‘AINO’—AINO enabled without MAR; and (4) AINO with MAR enabled. Each post-implant configuration (C1 and C2) was scanned four times under these reconstruction settings, resulting in a total of 33 CBCT scans (i.e., 1 for C0, 4 × 4 for C1, and 4 × 4 for C2).

### 2.2. Image Analysis

Although image registration and ROI placement may resemble steps used in qualitative or visual assessments, in this study they served exclusively to standardize voxel correspondence for quantitative gray-value extraction. Once registered, all ROIs were automatically applied to every scan using an ImageJ v1.54p macro, ensuring identical sampling locations. No visual scoring, subjective evaluation, or categorical grading of artifact severity was performed. All outcomes consisted of continuous numerical gray-value measurements derived from fixed-size ROIs across five axial slices, which were subsequently analyzed using statistical tests. Therefore, the study design and data structure are fundamentally quantitative in nature.

#### 2.2.1. Image Registration

All scans were aligned with the pre-implant scan (C0) to ensure exact voxel-level reproducibility of the region of interest. Registrations were performed in elastix https://github.com/SuperElastix/elastix (accessed on 9 July 2025) using the following parameters:

Registration mode: Multi-resolution registration (number of resolution = 4)

Transform: Euler transform

Interpolation: B-spline

Optimizer: Adaptive stochastic gradient descent (maximum iterations = 5000)

Registration metric: Advanced Mattes Mutual Information.

For each registration, a ROI was defined in the vicinity of the implants to avoid any effect of tissues that may have moved non-rigidly throughout the scans (i.e., soft tissue, upper jaw). Registered scans were saved in Tag Image File format for further analysis.

#### 2.2.2. Gray Value Normalization

The gray value distribution for all scans was rescaled to ensure consistency between comparative gray value measurements performed in the next step. A macro was written in ImageJ to automate this procedure. A linear ROI through cortical bone and a small rectangular ROI in the soft tissue were defined at an axial level below that of the implants. The mean gray value in both ROIs was measured for all scans. Subsequently, using the ROI values measured for the pre-implant scan as calibration points, the values for all other scans i were adjusted as follows:

$${GV}_{new,i}=\left({GV}_{old,i}-{MGV}_{soft,i}\right)\times \frac{\left({MGV}_{bone,0}-{MGV}_{soft,0}\right)}{\left({MGV}_{bone,i}-{MGV}_{soft,i}\right)}\;+{MGV}_{soft,0}$$

with *GV* denoting the gray value for each voxel, *MGV* the mean gray value for the abovementioned calibration ROIs (soft tissue and bone), and the index 0 corresponding to the pre-implant scan.

#### 2.2.3. Artifact Region of Interest Selection

Regions of interest around the implants were defined by researchers. As all scans were registered, the ROI was identified on one post-implant scan and then saved and applied to all other scans using a fully automated ImageJ script. For each ROI, the mean gray value was measured in five consecutive axial slices.

Criteria for the ROIs were

1. Rectangular, equal width and height (10 × 10 voxels);
2. Close to the implants, but not containing any part of the implant itself.

**Figure 2:**
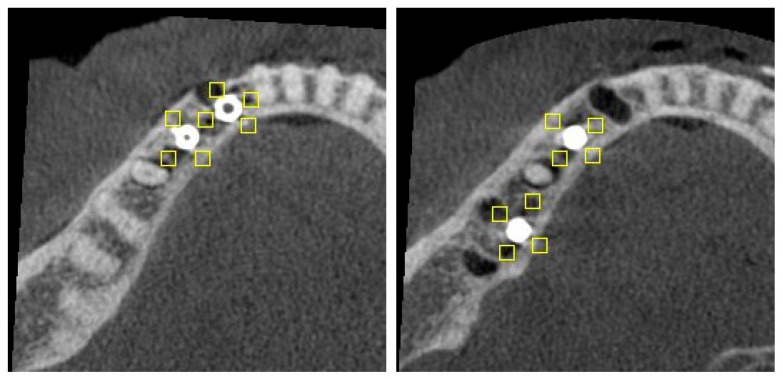
Axial slice of C1 (**left**) and C2 (**right**) configurations, showing position of ROIs (rectangles).

For the two implant conditions, ROIs were placed as follows (Figure 2):

- C1—lingual, buccal and mesial to the mesial implant; lingual, buccal and distal to the distal implant; and an additional ROI between the implants (n = 7).
- C2—lingual, buccal, mesial and distal to each implant (n = 8).

### 2.3. Statistical Analysis

The distributions of gray values were examined by Shapiro–Wilk’s test, normality plots and skewness/kurtosis statistics. Gray values were summarized by the mean ± standard deviation and median with an interquartile range.

The set of scans within C1 and C2 implant configurations were compared by repeated measures ANOVA with Greenhouse-Geisser correction. Bonferroni post hoc test was performed for pairwise comparisons. The gray values of each pair of scans were compared by either independent samples *t*-test and Mann–Whitney U test between C0 and OFF configurations, and by paired samples *t*-test and Wilcoxon test between the four scan conditions (OFF, MAR, AINO, and AINO+MAR) considering the distribution of gray values. A *p*-value ≤ 0.05 was considered statistically significant.

All statistical analyses were performed via IBM SPSS Statistics 22.0 (IBM Corp. Released 2013. IBM SPSS Statistics for Windows, Version 22.0. IBM Corp., Armonk, NY, USA).

For each implant configuration (C1 and C2), pairwise comparisons were conducted across the four reconstruction modes (OFF, MAR, AINO, and AINO+MAR). This resulted in six pairwise comparisons per configuration (OFF vs. MAR, OFF vs. AINO, OFF vs. AINO+MAR, MAR vs. AINO, MAR vs. AINO+MAR, AINO vs. AINO+MAR). Bonferroni correction was applied to control for multiple comparisons within each configuration.

Because this study used a single cadaveric specimen, the gray-value measurements obtained from multiple ROIs across consecutive slices represent technical replicates rather than biological replicates. Accordingly, the statistical comparisons reflect within-specimen differences between reconstruction modes and implant configurations, rather than biological population variability. The number of samples (*n*) for each analysis therefore corresponds to the number of ROI-based gray-value measurements rather than the number of biological specimens.

## 3. Results

**Figure 3:**
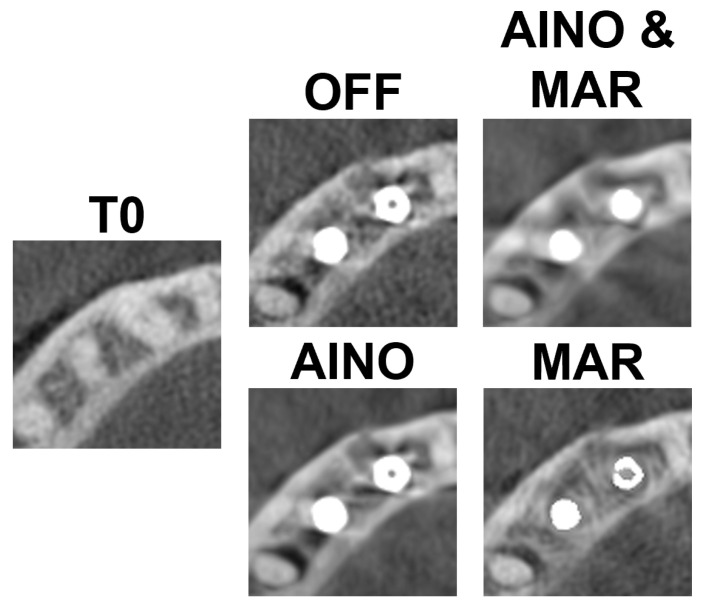
C1 configuration for each scan mode.

**Figure 4:**
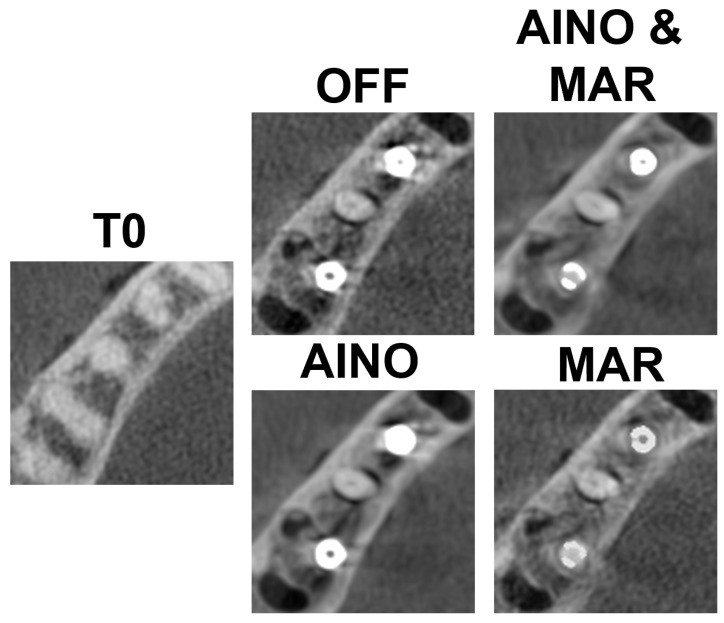
C2 configuration for each scan mode.

Figure 3 and Figure 4 present each implant and scan condition after registration and gray value rescaling at a fixed window/level. For the C1 configuration, the lowest gray values were in the inter-implant region and distal to the distal implant. For the C2 configuration, the lowest values were distal of the distal implant (Table 1). The registration was performed at a 32-bit depth, causing high overall values, so all values were divided by 105 for convenience. These values are not (pseudo-)Hounsfield units.

Table 2 presents the results per scan mode for the C1 configuration, compared with C0. There was no statistically significant difference in the lingual ROIs of the implants in the comparison of C1 OFF and control group (C0). In the comparison of C1 OFF and AINO, there was a statistically significant difference only in the distal part of the distal implant. Comparisons of AINO with AINO+MAR for C1 were only statistically significant for the distal ROI of the distal implant.

Table 3 presents the results per scan mode for the C2 configuration, compared with C0. Statistical significance was found in all ROIs. In the comparison of C2 OFF and AINO, statistical significance was found in the buccal and distal ROIs of the mesial implant, and in the buccal and lingual ROIs of the distal implant. In the comparison of AINO and AINO+MAR, all ROIs except the one buccal to the distal implant were significantly different (Table 3).

## 4. Discussion

In this study, the effect of MAR and AINO on gray values around implants was assessed ex vivo, yet in clinically realistic conditions. Although there are many studies in the literature using these algorithms, there is no study involving a fresh cadaver or assessing how the implant positions affect artifact formation [19,20,21,22,23,24,25]. The use of a complete cadaver head ensures appropriate depiction of soft and hard tissues and results in levels of attenuation, scatter, and beam hardening consistent with those of a patient.

Scans were performed using the largest available field of view (FOV) to control for gray value variability. Although smaller FOVs typically offer higher spatial resolution, our priority was reducing truncation artifacts and maintaining gray value consistency [26,27,28,29]. It is worth noting that larger FOVs introduce more scatter, which may destabilize gray values across the image [30,31,32]. While FOV effects on MAR have been previously investigated, fewer studies have focused on how AINO operates under varying acquisition parameters [33,34,35].

We employed a single CBCT device to ensure compatibility with the AINO filter, specific to this manufacturer’s reconstruction software. Although this limits generalizability, it provided control over reconstruction settings. Previous studies have confirmed that device-dependent variations significantly affect artifact severity and image quality [36,37].

Prior research has evaluated MAR and AINO at a diagnostic level. Kurt et al. evaluated the image quality in a study in which 4 different CBCT scans (MAR−/AINO−, MAR+/AINO−, MAR−/AINO+, MAR+/AINO+) were performed on a sheep mandible with artificial defects around zirconium and titanium implants [20]. The study observed that both the MAR module and AINO filter increased the accuracy of the detection of peri-implant fenestrations. Shahmirzadi et al. showed MAR reduced artifacts with all tested implant materials. Parsa et al. observed differences between images with and without MAR, though not always significant [23,24].

Shahmirzadi et al. assessed beam hardening artifacts in CBCT images using MAR before exposure, after exposure, and no MAR with three implant materials (Titanium-Zirconium, Titanium, and Zirconium alloy) at 84 and 90 kV. They found fewer artifacts at 90 kV. They also noted that images with MAR before or after irradiation had fewer artifacts than those without MAR, and it is not crucial whether MAR is applied before or after the scan, as it does not impact exposure [23].

A study by Parsa et al. evaluated the artifacts in CBCT images for three different conditions (pre-implant/post-implant without MAR/post-implant with MAR) [24]. They found a significant difference between the images without implants and the other two conditions, similar to the present study. Unlike the present study, however, no significant difference was found between MAR ON and MAR OFF modes after the implant was placed.

Fontenele et al., in their study, investigated the effect of tube current and MAR activation on the diagnosis of vertical fracture in the tooth adjacent to the implant on CBCT images. They found that neither tube current nor MAR activation had a significant effect on the diagnosis of vertical root fracture [38]. In our current study, it was found that MAR activation was significantly affected by implant position. Therefore, MAR activation did not cause a significant change in the images in the C1 configuration, which is the implant configuration where two implants are side by side, while MAR activation in the C2 configuration, which is the tooth configuration between the implants, showed a significant difference in the images.

In another CBCT study investigating the effect of MAR on the determination of dehiscence and fenestration around dental implants, similar to the C1 implant configuration used in this study, it was found that MAR did not have a substantial effect on image quality [39].

Bayrak et al. and Fontenele et al. demonstrated limited diagnostic improvement with MAR in adjacent implant scenarios, aligning with our C1 findings. In contrast, the C2 configuration showed substantial artifact reduction, supporting the benefit of combining MAR and AINO in clinical conditions where implants are spaced apart. Based on this, it can be interpreted that the application of combined MAR and AINO may be affected by the position of the implants. In addition, Schulze highlighted more pronounced artifacts for titanium implants. Vasconcelos et al. found MAR more effective near zirconia implants compared to titanium. Our study focused exclusively on titanium to minimize confounding variables, [40]. whereas Vasconcelos et al. showed that a proprietary MAR algorithm was more effective in improving the contrast-to-noise ratio in regions around zirconia implants compared with titanium implants [41]. The fact that our study evaluated only titanium as an implant material can be considered a limitation, as we opted to use one type of implant material and surgical procedure to avoid introducing additional variables (other than implant configuration and reconstruction/post-processing) that could affect the depiction of artifacts. Furthermore, our study focused on gray value effects rather than diagnostic benefits of MAR and/or AINO. While this study allowed for an objective image quality analysis, future research should focus primarily on the effect (or lack thereof) of reconstruction and post-processing algorithms on the detection of pathology and other diagnostic metrics.

This study hypothesized that implant configuration affects the severity of beam hardening artifacts, and the application of AINO, either alone or in combination with MAR, would reduce artifacts more effectively than standard reconstruction. Both hypotheses were supported. Artifact severity was significantly lower in the C2 configuration compared to C1, and the combined application of MAR and AINO in C2 led to gray values that closely approximated those of the artifact-free baseline (C0).

These findings have important clinical implications. Artifact-induced gray value distortions may lead to misinterpretation of peri-implant tissues, potentially simulating bone loss or concealing real defects. Our data suggest that post-processing filters like MAR and AINO can improve diagnostic reliability, particularly when implants are not positioned adjacently. For clinicians, this highlights the need to account for implant layout during image interpretation and to use available filtering options strategically.

Although the study employed voxel-level registration, automated ROI transfer, and gray-value rescaling to standardize measurements, several methodological limitations should be acknowledged. First, all measurements were obtained from a single cadaveric specimen, meaning that the ROI-based gray values represent technical rather than biological replicates, and therefore do not capture inter-individual variability. In addition, while Bonferroni correction was applied to control for type I error across the multiple pairwise comparisons, these statistical tests reflect within-specimen technical consistency rather than population-level generalizability. As a result, the interpretation of *p*-values is inherently limited in this context. Accordingly, effect sizes, absolute gray-value differences, and gray-value recovery ratios relative to the implant-free reference (C0) provide more meaningful insight into artifact behavior. Future studies incorporating multiple specimens, repeated measurements, and standardized calibration phantoms are necessary to enhance reproducibility and support more robust quantitative inference.

## 5. Conclusions

This study demonstrates that implant positioning plays a decisive role in the severity and pattern of beam hardening artifacts in CBCT imaging, and that the usefulness of MAR and AINO is highly dependent on these geometric relationships. Clinically, these findings are important because artifact-related gray value distortions can obscure buccal or lingual cortical contours, mimic peri-implant bone loss, or mask early signs of dehiscence and fenestration. Such misinterpretations may delay the diagnosis of peri-implant pathology or lead to incorrect assumptions regarding osseointegration.

Our results indicate that when implants are placed adjacent to one another (C1 configuration), the accumulation of dense metal structures produces intensified photon starvation and streaking artifacts that are minimally corrected by MAR or AINO. In contrast, when implants are separated by a natural tooth (C2 configuration), the combined use of MAR and AINO restores gray values closer to the artifact-free baseline, improving visualization of peri-implant bone and potentially enhancing diagnostic confidence.

These findings underscore the clinical value of considering implant spacing not only during treatment planning but also when interpreting postoperative CBCT scans. They further suggest that artifact-reduction tools should be used strategically, with greatest expected benefit in configurations where metal interactions are reduced. Future studies should assess how these gray-value improvements translate to diagnostic accuracy in detecting peri-implant defects and pathology.

## Figures and Tables

**Table 1:** Comparison of average rescaled gray values measured in specific ROIs for C1 (adjacent implants) and C2 (implants with intervening tooth) configurations to evaluate how implant positioning affects artifact severity.

Region of Interest	C1	C2
	(Mean ± SD)	(Mean ± SD)
Between implants	1.885 ± 0.316	NA
Distal implant		
Lingual side	2.690 ± 0.484	2.621 ± 0.069
Buccal side	3.273 ± 0.171	1.397 ± 0.106
Distal side	1.863 ± 0.144	1.227 ± 0.069
Mesial side	NA	1.489 ± 0.101
Mesial implant		
Lingual side	2.906 ± 0.266	2.957 ± 0.105
Buccal side	2.920 ± 0.497	2.987 ± 0.099
Distal side	NA	1.808 ± 0.053
Mesial side	2.762 ± 0.235	2.875 ± 0.080

SD: Standard deviation, NA: Not applicable.

**Table 2:** Comparison of the mean gray values across different scan conditions (OFF, AINO, MAR, AINO+MAR) for the C1 configuration, to evaluate the effectiveness of artifact reduction techniques in regions surrounding adjacent implants.

Region of Interest	C0	OFF	AINO+	AINO+MAR high	*p*-Value ^1^	*p*-Value ^2^	*p*-Value ^3^
	Mean ± SD	Mean ± SD	Mean ± SD	Mean ± SD			
Between implant	1.885 ± 0.316	2.283 ± 0.082	1.674 ± 0.422	2.214 ± 0.214	0.012	<0.001	0.001
Median (IQR)	1.850 (1.669–2.178)	2.296 (2.204–2.356)	1.510 (1.373–2.169)	2.186 (2.027–2.361)			
Distal implant							
Lingual	2.690 ± 0.484	3.089 ± 0.059	2.939 ± 0.444	3.126 ± 0.113	0.575	0.156	>0.999
Median (IQR)	2.795 (2.249–3.161)	3.122 (3.025–3.136)	3.104 (2.488–3.245)	3.118 (3.016–3.238)			
Buccal	3.273 ± 0.171	3.978 ± 0.082	3.323 ± 0.103	3.354 ± 0.155	<0.001	0.371	0.154
Median (IQR)	3.306 (3.253–3.370)	4.003 (3.913–4.030)	3.344 (3.205–3.424)	3.401 (3.234–3.463)			
Distal	1.863 ± 0.144	2.129 ± 0.184	1.985 ± 0.193	2.275 ± 0.183	0.006	0.040	0.001
Median (IQR)	1.884 (1.724–1.995)	2.065 (1.990–2.300)	1.978 (1.854–2.167)	2.257 (2.121–2.419)			
Mesial implant							
Lingual	2.906 ± 0.266	2.991 ± 0.025	2.837 ± 0.249	2.682 ± 0.387	0.717	0.575	0.062
Median (IQR)	2.849 (2.682–3.183)	2.983 (2.969–3.016)	2.878 (2.634–3.058)	2.649 (2.337–3.096)			
Buccal	2.920 ± 0.497	4.402 ± 0.060	2.700 ± 0.306	2.742 ± 0.290	<0.001	0.575	0.137
Median (IQR)	2.788 (2.497–3.399)	4.388 (4.350–4.462)	2.581 (2.481–2.976)	2.614 (2.543–3.028)			
Mesial	2.762 ± 0.235	2.464 ± 0.134	2.713 ± 0.128	2.684 ± 0.123	0.015	0.550	0.482
Median (IQR)	2.780 (2.558–2.998)	2.381 (2.373–2.596)	2.726 (2.613–2.789)	2.663 (2.588–2.749)			

Gray values were summarized by mean ± standard deviation and median (IQR). ^1^ C0 vs. C1 OFF, ^2^ C0 vs. AINO+ comparison, ^3^ AINO+ vs. AINO+MAR high comparison.

**Table 3:** Comparison of the mean gray values under various scan conditions (OFF, AINO, MAR and AINO+MAR) for the C2 configuration, to assess the performance of artifact reduction techniques around implants placed with an intervening tooth.

Region of Interest	C0	OFF	AINO+	AINO+MAR high	*p*-Value ^1^	*p*-Value ^2^	*p*-Value ^3^
	Mean ± SD	Mean ± SD	Mean ± SD	Mean ± SD			
Distal implant							
Lingual	2.621 ± 0.069	2.409 ± 0.029	2.583 ± 0.031	2.361 ± 0.055	<0.001	0.005	<0.001
Median (IQR)	2.643 (2.569–2.662)	2.404 (2.388–2.433)	2.587 (2.566–2.609)	2.359 (2.322–2.409)			
Buccal	1.397 ± 0.106	3.567 ± 0.117	1.434 ± 0.086	1.495 ± 0.135	<0.001	0.006	0.067
Median (IQR)	1.398 (1.339–1.498)	3.496 (3.477–3.691)	1.453 (1.359–1.489)	1.438 (1.413–1.556)			
Distal	1.227 ± 0.069	1.698 ± 0.163	1.227 ± 0.035	1.744 ± 0.106	<0.001	0.989	<0.001
Median (IQR)	1.222 (1.185–1.277)	1.674 (1.546–1.862)	1.223 (1.202–1.247)	1.764 (1.669–1.834)			
Mesial	1.489 ± 0.101	2.001 ± 0.081	1.477 ± 0.096	1.838 ± 0.114	<0.001	0.267	<0.001
Median (IQR)	1.483 (1.420–1.575)	1.968 (1.932–2.087)	1.487 (1.426–1.537)	1.841 (1.798–1.899)			
Mesial implant							
Lingual	2.957 ± 0.105	2.787 ± 0.096	2.942 ± 0.094	2.751 ± 0.132	<0.001	0.209	<0.001
Median (IQR)	2.974 (2.945–3.014)	2.769 (2.702–2.881)	2.946 (2.890–3.011)	2.741 (2.656–2.820)			
Buccal	2.987 ± 0.099	3.428 ± 0.128	3.023 ± 0.098	2.953 ± 0.168	<0.001	0.003	0.001
Median (IQR)	2.992 (2.927–3.075)	3.398 (3.312–3.560)	3.006 (2.949–3.092)	2.936 (2.814–3.068)			
Distal	1.808 ± 0.053	2.259 ± 0.088	1.776 ± 0.044	2.304 ± 0.071	<0.001	0.029	<0.001
Median (IQR)	1.803 (1.786–1.847)	2.283 (2.185–2.321)	1.774 (1.751–1.801)	2.313 (2.245–2.373)			
Mesial	2.875 ± 0.080	2.406 ± 0.042	2.864 ± 0.070	2.519 ± 0.140	<0.001	0.353	<0.001
Median (IQR)	2.886 (2.831–2.924)	2.416 (2.368–2.440)	2.869 (2.827–2.903)	2.559 (2.404–2.636)			

^1^ C0 vs. C2 OFF comparison, ^2^ C0 vs. AINO+ comparison, ^3^ AINO+ vs. AINO+MAR high comparison.

## Data Availability

The datasets used and/or analyzed during the current study available from the corresponding author on reasonable request.
